# The performance of anti–cyclic citrullinated peptide antibodies in predicting the severity of radiologic damage in inflammatory polyarthritis: Results from the Norfolk Arthritis Register

**DOI:** 10.1002/art.22868

**Published:** 2007-09

**Authors:** M Bukhari, W Thomson, H Naseem, D Bunn, A Silman, D Symmons, A Barton

**Affiliations:** 1 University of ManchesterManchester, UK; 2 Norfolk Arthritis Register, and Norfolk and Norwich HospitalNorfolk, UK

## Abstract

**Objective:**

Anti–cyclic citrullinated peptide (anti-CCP) antibodies are a stronger predictor of the severity of rheumatoid arthritis than is rheumatoid factor (RF). Their role in predicting outcome in unselected patients with new-onset inflammatory polyarthritis (IP) has not been examined. The aims of this study were to examine the role of baseline RF and anti-CCP antibodies in determining the likelihood of patients having erosions at presentation or in predicting future radiologic damage, and to determine whether anti-CCP antibodies or RF is sufficiently robust to be clinically useful in guiding treatment decisions in early IP.

**Methods:**

Patients were recruited from the Norfolk Arthritis Register. Logistic regression models were fitted to test the ability of anti-CCP antibodies and RF to predict erosions. Further models were investigated to examine the role of anti-CCP antibodies in patients stratified by RF status.

**Results:**

The presence of anti-CCP antibodies at baseline was strongly associated with both prevalent erosions (odds ratio [OR] 2.53 [95% confidence interval (95% CI) 1.48–4.30]) and developing erosions at 5 years (OR 10.2 [95% CI 6.2–16.9]). These ORs were higher than those for RF (OR 1.63 [95% CI 0.94–2.82] and OR 3.4 [95% CI 2.2–5.2], respectively). The likelihood ratio (LR) for the prediction of prevalent erosions and erosions at 5 years was highest in the RF−subgroup (LR 2.2 and 5.8, respectively). However, 27% of anti-CCP−patients had developed erosions by 5 years.

**Conclusion:**

Despite their strong association with the presence, development, and extent of erosions, anti-CCP antibodies alone are not a sufficiently accurate measure upon which to base clinical treatment decisions. Knowledge of anti-CCP antibody status is most informative in RF−negative patients.

There is considerable evidence of the benefit of early treatment with disease-modifying antirheumatic drugs (DMARDs) in patients with rheumatoid arthritis (RA). These studies have shown that there is a window of opportunity early in the disease course during which DMARDs have the greatest effect in altering disease progression, as measured by the development of radiologic erosions (1,2). The identification of a marker at the onset of disease that could reliably predict which patients will or, perhaps more importantly, will not develop erosions would be a major clinical advance because the latter group could be spared potentially toxic therapies, while the former group may be targeted for combination or biologic therapy. There have been several prospective studies that have examined the relative role of different clinical and laboratory predictors. The presence of rheumatoid factor (RF) and of shared epitope (SE) alleles of the *HLA–DRB1* gene has been consistently associated with an adverse outcome (3–5).

More recently, studies have has focused on the role of antibodies that recognize cyclic citrullinated peptides (anti–citrullinated protein antibodies [ACPAs]) (6). ACPAs, as measured by anti–CCP-2 enzyme-linked immunosorbent assays (ELISAs), are highly specific and reasonably sensitive for diagnosing RA (7), although RF may still be present in persons with RA who are negative for anti-CCP antibodies (8). Cross-sectional surveys of prevalent RA cases have also shown that both RF and anti-CCP antibodies are associated with radiographic severity (6,9–11), but recent studies suggest that their effects are not completely overlapping (10). Prospective studies have confirmed the association of anti-CCP antibodies with worsening radiographic outcome in patients with RA at baseline (6,12–18). It has also been shown that the presence of these antibodies in patients presenting with undifferentiated inflammatory arthritis is associated with an increased likelihood of being classified as having RA (13).

A limitation of many previous studies has been the restriction of the investigation to patients with definite RA. In this group, it is difficult to evaluate erosions as an outcome since erosions are one of the criteria used for classification of RA. We have argued previously that an unselected series of patients with inflammatory polyarthritis (IP) would provide a more representative cohort in which to develop prognostic models because the American College of Rheumatology (ACR; formerly, the American Rheumatism Association) classification criteria for RA (19) do not perform well in early disease (20). Furthermore, rheumatologists increasingly want to make therapeutic decisions before patients satisfy the ACR criteria, because there is considerable evidence to suggest that it is in the early stages of the disease that treatment is most likely to affect outcome (21).

In our previous study of primary care–based unselected series of patients with IP presenting between 1990 and 1994, we reported that RF was the most important baseline predictor of erosive disease at 5 years (22), but we have not examined the role of anti-CCP antibodies. The aim of this study was to compare the roles of RF and anti-CCP antibody status in determining the likelihood of having erosions at presentation and in predicting future radiologic damage. We also determined whether anti-CCP antibody status, either alone or in combination with RF, is sufficiently robust to be useful in guiding clinical treatment decisions.

## PATIENTS AND METHODS

### Study protocol

Subjects were recruited from the Norfolk Arthritis Register (NOAR), a primary care–based inception cohort of patients with IP. Details of NOAR have been published previously (23). Briefly, patients with swelling in 2 or more joints that lasted 4 weeks or longer were referred to NOAR and were assessed by a trained metrologist within 2 weeks of referral using a standardized approach. Data gathered included joint counts for swelling and tenderness and responses on the Health Assessment Questionnaire (24). Blood was obtained for serum analysis (initially for RF, but C-reactive protein and anti-CCP testing have been introduced more recently) and DNA extraction.

Radiographs of the hands and feet were requested for each consenting patient and were scored using the Larsen scale (25). All radiographs were scored by 2 observers, with a third observer arbitrating in case of disagreement (MB, DS, and AB). Details of the radiographic scoring process used in NOAR have been published elsewhere (22). Briefly, a Larsen score of ≥2 in any joint indicated the presence of erosions. Joints assessed include all proximal interphalangeal joints, the interphalangeal joint of the thumbs, all metacarpophalangeal joints, both wrists, and the second through fifth metatarsophalangeal joints in both feet. A weighting factor of 5 was applied to each wrist. The total possible score was 190.

Criteria for ascertainment of radiographs have changed over the time period that patients have been recruited to NOAR. Two cohorts of patients were included in the current study. The prospective cohort was composed of 427 consecutive unselected patients recruited between 1990 and 1994 who had both a baseline serum sample and a radiograph at 5 years available for analysis. These patients did not have a baseline radiograph performed. A more recently recruited cross-sectional cohort was studied based on 254 consecutive patients recruited after January 1, 2000 who had a baseline radiograph performed.

### Serum testing

RF was measured using a latex method, and a titer of ≥1:40 was regarded as positive. Anti-CCP testing was performed using the Axis-Shield DIASTAT kit according to the manufacturer's instructions (Axis-Shield, Dundee, UK), using the recommended cutoff of >5 units/ml as positive.

### Statistical analysis

The baseline characteristics of the cohorts were compared, stratified according to their anti-CCP and RF status. For the prospective cohort, erosion status and Larsen score at 5 years were also analyzed in this way. Categorical values were compared using the chi-square test, and continuous variables were compared using the Mann-Whitney U test.

We constructed 2 × 2 tables examining the prevalence of erosions in the various groups, depending on their anti-CCP and RF status alone and in combination. Odds ratios (ORs) and 95% confidence intervals (95% CIs) were calculated using Stata software (StataCorp, College Station, TX). The absolute values of ELISA-measured anti-CCP antibody levels were also used as a continuous variable to determine their association with the development of erosions at 5 years using a receiver operating curve (ROC) analysis.

Four groups were identified within each cohort, depending on their autoantibody profile: patients who were both anti-CCP+ and RF+, patients who were RF+ and anti-CCP+, patients who were RF− and anti-CCP+, and those who were both anti-CCP− and RF−. Larsen scores for subjects with erosions in the 4 groups were ascertained and compared using the Mann-Whitney U test. Sensitivity, specificity, and likelihood ratios (LRs) were calculated for each of these groups using the “diagt” command in Stata.

All analyses were repeated, adjusting for the use of DMARDs or steroids at 5 years. *P* values less than 0.05 were considered significant.

## RESULTS

### Clinical characteristics at baseline

The baseline characteristics of each cohort are shown in Table 1. At 5 years, 311 subjects in the prospective cohort (72.8%) had satisfied the ACR criteria for RA, modified for genetics studies. In the cross-sectional cohort, 88 (34.6%) were anti-CCP+, 71 (28.0%) were RF+, and 50 (19.7%) were anti-CCP+ and RF+, while in the prospective cohort, 125 (29.3%) were anti-CCP+, 113 (26.5%) were RF+, and 80 (18.7%) were anti-CCP+ and RF+. The presence of anti-CCP antibodies was highly, but not perfectly, correlated with the presence of RF. For example, the kappa statistic was 0.47 in the cross-sectional cohort (*P* < 0.001) and 0.59 in the prospective cohort (*P* < 0.001). The presence of anti-CCP antibodies, but not RF, at baseline was associated with prevalent erosions, and both were associated with erosions and was the presence of Larsen score by 5 years (Table 2).

**Table 1:** Patient characteristics at baseline and at 5 years, in patients with available data*

	Cross-sectional cohort (n = 254)	Prospective cohort (n = 427)
Baseline		
Female, no. (%)	173 (68.1)	283 (66.3)
Age at symptom onset, median (IQR) years	59.2 (48.4–70.6)	53.3 (42.6–70.5)
HAQ score, median (IQR)	0.88 (0.25–1.50)	0.75 (0.25–1.38)
RF+, no. (%)	71 (28.0)	113 (26.5)
Anti-CCP+, no. (%)	88 (34.6)	125 (29.3)
No. of tender joints, median (IQR)	3 (1–8)	8 (3–16)
No. of swollen joints, median (IQR)	3 (1–8)	6 (2–14)
Shared epitope alleles, no. (%)		
0	79 (48.5)	155 (39.4)
1	63 (38.6)	181 (46.1)
2	21 (12.9)	57 (14.5)
5 years		
No. of tender joints, median (IQR)	–	0 (0–4)
No. of swollen joints, median (IQR)	–	0 (0–2)
HAQ score, median (IQR)	–	0.75 (0.25–1.5)
Treated with DMARD or steroid by 5 years, no. (%)	–	257 (60.2)
Satisfied ACR criteria for RA by year 5, no. (%)	–	311 (72.8)
Symptom duration at baseline, median (IQR) months	5 (3–10)	5 (2–12)

* IQR = interquartile range; HAQ = Health Assessment Questionnaire; RF = rheumatoid factor; anti-CCP = anti–cyclic citrullinated peptide antibody; DMARD = disease-modifying antirheumatic drug; ACR = American College of Rheumatology; RA = rheumatoid arthritis.

**Table 2:** Clinical characteristics of the patients at baseline, according to the presence and absence of anti-CCP and RF at baseline*

	Anti-CCP+	Anti-CCP−	RF+	RF−
Cross-sectional cohort				
No. of subjects	88	166	71	183
Female, no. (%)	64 (72.7)	109 (65.7)	50 (70.4)	90 (67.2)
Age at symptom onset, median (IQR) years	60.7 (52.2–71.4)	58.5 (45.0–71.1)	62.7 (48.9–72.0)	58.5 (47.7–71.1)
HAQ score at baseline, median (IQR)	1.0 (0.38–1.75)	0.75 (0.25–1.50)†	1 (0.38–1.5)	0.88 (0.25–1.50)
Erosions at baseline, no. (%)	55 (62.5)	66 (39.8)‡	40 (56.3)	81 (44.3)
Larsen score at baseline, median (IQR)	6.5 (1–14.5)	2 (0–10)§	5 (0–15)	3 (0–12)
Prospective cohort				
No. of subjects	125	302	112	308
Female, no. (%)	77 (61.6)	206 (68.2)	71 (62.8)	212 (67.5)
Age at symptom onset, median (IQR) years	55.7 (48.4–64.0)	51.7 (40.8–62.7)	55.7 (46.8–64.0)	52.4 (42.1–62.4)
HAQ score, median (IQR)	0.88 (0.38–1.62)	0.63 (0.25–1.25)¶	0.75 (0.38–1.50)	0.75 (0.25–1.25)#
Erosions at 5 years, no. (%)	99 (79.2)	82 (27.2)**	74 (65.5)	107 (34.1)††
Larsen score at 5 years, median (IQR)	29 (12–44)	2 (0–10)¶	17 (3–41)	4 (0–14)¶
Treated with DMARD or steroid by 5 years, no. (%)	119 (95.2)	138 (45.7)‡‡	91 (80.5)	166 (52.9)§§

* See Table 1 for definitions.

† *P* = 0.04 versus anti-CCP+ group.

‡ *P* = 0.001 versus anti-CCP+ group.

§ *P* = 0.02 versus anti-CCP+ group.

¶ *P* < 1 × 10^−4^ versus anti-CCP+ group.

# *P* = 0.01 versus RF+ group.

** *P* = 4 × 10^−23^ versus anti-CCP+ group.

†† *P* = 6.7 × 10^−9^ versus RF+ group.

‡‡ *P* = 1.9 × 10^−21^ versus anti-CCP+ group.

§§ *P* = 2.6 × 10^−7^ versus RF+ group.

The presence of anti-CCP antibodies at baseline was a more powerful predictor of both prevalent erosions (OR 2.53 [95% CI 1.48–4.30]) and developing erosions by 5 years (OR 10.2 [95% CI 6.2–16.9]) than was the presence of RF (OR 1.63 [95% CI 0.94–2.82] and 3.4 [2.2–5.2], respectively) (Table 3). Modeling the role of anti-CCP antibodies stratified by RF status showed that anti-CCP antibodies perform equally well in predicting erosions in RF+ and RF− patients, and the presence of both autoantibodies did not yield significantly higher odds of erosions (either prevalent or at 5 years) than did anti-CCP alone (OR for erosions at 5 years in anti–CCP+ patients 10.2 [95% CI 6.2–16.9]; OR for erosions at 5 years in anti-CCP+, RF+ patients 11.6 [95% CI 4.5–29.9]).

**Table 3:** Prevalence and odds of erosions in patients at presentation and at 5 years, by autoantibody status at baseline*

	Antibody status at baseline				
	Present		Absent		
	No. (%) with erosions	No. (%) without erosions	No. (%) with erosions	No. (%) without erosions	OR (95% CI)
Cross-sectional cohort (at presentation)					
All anti-CCP+	55 (63)	33 (37)	66 (40)	100 (60)	2.53 (1.48–4.30)
Anti-CCP+, RF−	24 (63)	14 (37)	57 (39)	88 (61)	2.65 (1.27–5.54)
Anti-CCP+, RF+	31 (62)	19 (38)	9 (43)	12 (57)	2.18 (0.77–6.13)
All RF+	40 (56)	31 (44)	81 (44)	102 (56)	1.63 (0.94–2.82)
RF+, anti-CCP−	9 (43)	12 (57)	57 (39)	88 (61)	1.16 (0.46–2.92)
RF+, anti-CCP+	31 (62)	19 (38)	24 (63)	14 (37)	0.95 (0.40–2.28)
Prospective cohort (at 5 years)					
All anti-CCP+	99 (79)	26 (21)	82 (27)	220 (73)	10.2 (6.2–16.9)
Anti-CCP+, RF−	34 (76)	11 (24)	73 (27)	196 (73)	8.3 (4.0–17.2)
Anti-CCP+, RF+	65 (81)	15 (19)	9 (27)	24 (73)	11.6 (4.5–29.9)
All RF+	83 (63)	48 (37)	122 (34)	240 (66)	3.4 (2.2–5.2)
RF+, anti-CCP−	9 (27)	24 (73)	73 (27)	196 (73)	1.01 (0.45–2.27)
RF+, anti-CCP+	65 (81)	15 (19)	34 (76)	11 (24)	1.40 (0.58–3.39)

* OR = odds ratio; 95% CI = 95% confidence interval (see Table 1 for other definitions).

**Figure 1:**
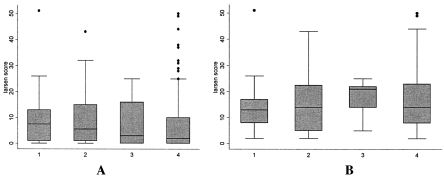
Distribution of Larsen scores at baseline in all subjects (**A**) and in subjects with prevalent erosions (**B**), according to baseline antibody status in the cross-sectional cohort. Values are presented as box and whisker plots, where the boxes represent the interquartile range, the lines within the boxes represent the median Larsen score, the whiskers represent the range from the smallest to the largest score, and the circles represent outliers. No significant differences between the groups were noted. 1 represents subjects who were RF+ and anti-CCP+ (*P* = 0.07 versus subjects who were RF− and anti-CCP− in the total group and *P* = 0.70 versus subjects who were RF− and anti-CCP− in the group with erosions). 2 represents subjects who were RF− and anti-CCP+ (*P* = 0.06 versus subjects who were RF− and anti-CCP− in the total group and *P* = 0.49 versus subjects who were RF− and anti-CCP− in the group with erosions). 3 represents subjects who were RF+ and anti-CCP− (*P* = 0.90 versus subjects who were RF− and anti-CCP− in the total group and *P* = 0.34 versus subjects who were RF− and anti-CCP− in the group with erosions). 4 represents subjects who were RF− and anti-CCP− (referent).

**Figure 2:**
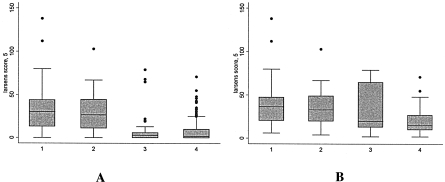
Distribution of Larsen scores at 5 years in all subjects (**A**) and in subjects with erosions (**B**), according to baseline antibody status in the prospective cohort. Values are presented as box and whisker plots, where the boxes represent the interquartile range, the lines within the boxes represent the median Larsen score, the whiskers represent the range from the smallest to the largest score, and the circles represent outliers. 1 represents subjects who were RF+ and anti-CCP+ (*P* < 0.0001 versus subjects who were RF− and anti-CCP−, both in the total group and in the group with erosions). 2 represents subjects who were RF− and anti-CCP+ (*P* < versus subjects who were RF− and anti-CCP− in the total group and *P* = 0.0001 versus subjects who were RF− and anti-CCP− in the group with erosions). 3 represents subjects who were RF+ and anti-CCP− (*P* = 0.90 versus subjects who were RF− and anti-CCP− in the total group and *P* = 0.85 versus subjects who were RF− and anti-CCP− in the group with erosions). 4 represents subjects who were RF− and anti-CCP− (referent).

Larsen scores were higher in anti-CCP+ patients than in anti-CCP− patients, both at baseline and at 5 years (Table 2). Patients with erosions who were anti-CCP+ at baseline had higher Larsen scores at 5 years (median 36, interquartile range [IQR] 20–48) than did anti-CCP− patients (median 16 [IQR 10–27]), although no difference in their baseline demographic features were noted. The median Larsen score at 5 years, but not at baseline, was higher in the anti-CCP+ patients, regardless of their RF status (Figures 1 and 2).

### Development of erosions

At 5 years, erosions had developed in 81% of RF+, anti-CCP+ patients and 27% of RF−, anti-CCP− patients. The titers of anti-CCP antibodies were higher in RF+ (median 25.9 [95% CI 1.0–74.3]) than in RF− (median 0.74 [95% CI 0.48–1.26]) patients. The sensitivity and specificity of the models derived from Table 3 showed that both RF and anti-CCP antibodies were stronger predictors of erosions at 5 years than at presentation (Table 4). The specificities for predicting erosions at 5 years were similar for RF and anti-CCP antibodies, but the latter were more sensitive. Interestingly, the presence of anti-CCP antibodies had the highest LR for erosions, whether at baseline or at 5 years, in RF− patients. However, the area under the curve in the ROC analysis was higher in RF+, anti-CCP+ subjects than in RF−, anti-CCP+ subjects (0.78 [95% CI 0.69–0.87] and 0.66 [95% CI 0.59–0.73], respectively), suggesting that anti-CCP is a better predictor of erosions at 5 years in the presence of RF positivity.

**Table 4:** Sensitivity, specificity, and likelihood ratios for predicting erosions at baseline or at 5 years using baseline RF and anti-CCP status either alone or in combination*

	Sensitivity, %	Specificity, %	Likelihood ratio
Cross-sectional cohort			
All anti-CCP+	45.5	75.2	1.8
Anti-CCP+, RF−	29.6	86.3	2.2
Anti-CCP+, RF+	77.5	38.7	1.3
All RF+	33.1	76.7	1.4
RF+, anti-CCP−	13.6	88.0	1.1
RF+, anti-CCP+	56.4	42.4	1.0
Prospective cohort			
All anti-CCP+	54.7	89.4	5.2
Anti-CCP+, in RF−	31.8	94.5	6.0
Anti-CCP+, in RF+	87.8	61.5	2.3
All RF+	40.9	85.2	2.4
RF+, anti-CCP−	11.0	89.1	1.0
RF+, anti-CCP+	65.7	42.3	1.1

* See Table 1 for definitions.

The ROC analysis was also used to determine whether there is a cutoff of anti-CCP antibody values at which erosions at 5 years can be accurately predicted. The peak of the ROC curve occurred at an anti-CCP antibody value of 2.4 when the whole data set was included and at 0.65 when analysis was restricted to RF− subjects, indicating that, even at low titers, the presence of the antibody is associated with erosive change.

Logistic regression analyses were repeated using DMARDs or steroids at 5 years as a cofactor, but in no situation did this correction alter the conclusions, although it did attenuate the effects seen. For example, the OR of developing erosions at 5 years in the presence of anti-CCP antibodies at baseline was reduced to 7.1 (95% CI 4.1–12.1) after accounting for this potential confounder.

## DISCUSSION

We have shown that anti-CCP antibody status, measured at presentation of IP, predicts both prevalent erosions and development of erosions at 5 years. Furthermore, anti-CCP antibody status is a better predictor of future erosions than is RF, despite the fact that both are correlated.

Anti-CCP antibody status has been proposed as a new biomarker of disease severity, since it has been found to be more sensitive than RF by all who have published studies on this area. These antibodies have the advantage that the status is stable over time as compared with RF, which is known to vary (26). Questions remain, however, regarding their usefulness in clinical practice, particularly because the assay cost is higher than that of RF, and it is unclear how much better than RF they are at determining outcome. It is unclear whether both RF and anti-CCP antibodies should be tested routinely in patients or whether anti-CCP antibody testing should be reserved for those who are RF negative.

Our study aimed to inform the debate and has several advantages over previous investigations. First, we used a primary care–based cohort of patients with unselected IP, thus reflecting the mix of patients attending early arthritis clinics. Second, the study design removes possible biases introduced when analysis is restricted to RA patients, because erosions and RF (with which anti-CCP antibodies are correlated) are both criteria used to classify RA. Finally, the radiographs were read blinded to anti-CCP status, removing the possibility of observer bias. We tested radiologic erosions as the primary outcome measure because they are an objective, reliable, and standardized method of measuring arthritis severity (27).

The results show that both RF and anti-CCP antibody status are useful as predictors of adverse outcome but that anti-CCP antibody status is stronger. The group of patients who were anti-CCP− but RF+ had similar Larsen scores as the group negative for both antibodies and significantly lower scores than the anti-CCP+, RF− group, although the small numbers in some of the groups may have limited the robustness of these conclusions. The LR for predicting erosions was higher in the anti-CCP+, RF− group, suggesting that one possible strategy would be to test for anti-CCP antibodies only in patients seronegative for RF.

It should be noted, however, that a significant number of anti-CCP− patients developed erosions at 5 years (27% of the prospective cohort). Larsen scores were significantly lower in these patients compared with anti-CCP+ subjects with erosions (median 16 [IQR 10–27] and 36 [IQR 20–48], respectively), suggesting that anti-CCP antibodies may affect the extent or severity of radiologic damage as well as susceptibility to it. However, the absence of anti-CCP antibodies cannot be used to identify subjects who do not require treatment, since even very low titers can be associated with the development of erosions, particularly in RF− subjects.

Conversely, 21% of patients positive for the presence of anti-CCP antibodies had not developed erosions at 5 years. This may reflect the benefit of treatment if treatment was more likely to be provided to anti-CCP+ patients, and indeed, this was found to be the case. Thus, although the treating physician was unaware of the antibody status, the presence of anti-CCP antibodies was strongly associated with the likelihood of receiving DMARDs or steroid therapy (OR 16.6 [95% CI 8.9–30.7]), presumably because of other markers of disease severity. The presence of RF was also associated with receiving treatment, but to a lesser degree (OR 5.2 [95% CI 3.4–7.8]). To address the issue of possible confounding by treatment, we adjusted for ever use of DMARDs or steroids at 5 years in the analysis. Such an adjustment is inevitably quite crude and almost certainly will not have accounted for all the treatment effects. The results, after this adjustment, showed that although the effects were attenuated, the presence of either antibody remained strongly predictive of the development of erosions.

In summary, ACPAs, as measured by anti-CCP ELISAs, are strongly associated with both prevalent erosions and the development of erosions at 5 years. In this respect, it is a stronger predictor than RF, but because its detection contributes no additional value in RF+ patients, testing could be restricted to seronegative patients. Finally, despite the strong association of anti-CCP antibodies with the presence, development, and extent of erosions, knowledge of anti-CCP status alone is still not a sufficiently accurate measure upon which to base clinical treatment decisions, since a significant proportion of anti-CCP− patients develop erosions.

## AUTHOR CONTRIBUTIONS

Dr. Bukhari had full access to all of the data in the study and takes responsibility for the integrity of the data and the accuracy of the data analysis.

**Study design.** Silman, Symmons, Barton.

**Acquisition of data.** Thomson, Naseem, Bunn, Symmons,

**Analysis and interpretation of data.** Bukhari, Naseem, Silman, Symmons, Barton.

**Manuscript preparation.** Bukhari, Thomson, Naseem, Silman, Symmons, Barton.

**Statistical analysis.** Bukhari, Naseem, Barton.
